# Analysis of the Mortality Trends of 23 Major Cancers in the Indian Population Between 2000 and 2019: A Joinpoint Regression Analysis

**DOI:** 10.1200/GO.22.00405

**Published:** 2023-03-22

**Authors:** Ajil Shaji, Pavithran Keechilat, Vijaykumar DK, Catherine Sauvaget

**Affiliations:** ^1^ Amrita Institute of Medical Sciences, Amrita VishwaVidhyapeedham, Cochin, India; ^2^ Early Detection, Prevention and Infections Branch, International Agency for Research on Cancer, Lyon, France

## Abstract

**PURPOSE:**

Cancer mortality trends have not been documented across the population of India. We, therefore, analyzed the overall and individual cancer mortality trends for 23 major cancers between 2000 and 2019 on the basis of Global Health Observatory (GHO) database.

**MATERIALS AND METHODS:**

This study examined cancer mortality trends for 23 major cancer sites on the basis of 12.85 million cancer deaths obtained from the GHO of the WHO between 2000 and 2019. A joinpoint regression model was used to analyze the long-term trends of cancer mortality. Annual percentage change (APC) and average APC were estimated for various cancer sites.

**RESULTS:**

Between 2000 and 2019, 12.85 million deaths occurred in India from 23 major cancers. The most common lethal cancers were mouth and oropharyngeal (15.6%), stomach (10.6%), lung (9.6%), breast (9%), and colorectal (8%) cancers. The mortality trend decreased by 0.19% annually among men and increased nonsignificantly by 0.25% among women; an increase of 0.02% was observed among combined sexes. Increasing mortality trends were seen among cancers of the lung, breast, colorectum, lymphoma, multiple myeloma, gallbladder, pancreas, kidney, and mesothelioma between 2000 and 2019. The highest annual increase in mortality was observed in pancreatic cancer among both sexes: 2.7%, 2.1% among men, and 3.7% in women. The cancers of the stomach, esophagus, leukemia, larynx, and melanoma showed a declining cancer mortality trend irrespective of sex.

**CONCLUSION:**

A multifaceted strategy is required to tackle the rising cancer mortality rates in India; the best long-term strategy could be implementing awareness on cancer symptoms among the population as well as cancer prevention policies with improved health infrastructure and specifically dedicated human resources.

## INTRODUCTION

Globally, cancer is the second most lethal noncommunicable disease after cardiovascular disease and accounted for about 9.9 million deaths in 2020. Around 9% of all cancer deaths occurred in the Indian population.^1,2^ Cancer registries are the primary resource for collecting information on cancer for understanding the magnitude, patterns, and trends over time, as well as for planning cancer control activities and patient care.^3,4^ The National Cancer Registry Programme (NCRP) was initiated by the Indian Council of Medical Research with three population-based cancer registries (PBCRs) and three hospital-based cancer registries (HBCRs) in India. The NCRP has performed cancer surveillance for the past four decades and, by 2020, it expanded to 36 PBCRs and 236 HBCRs covering around 10% of the country's total population.^5^

**CONTEXT**

**Key Objective**
How has the number of cancer-related fatalities changed in India over the past two decades?
**Knowledge Generated**
Cancer mortality trend among men in India have shown a slight yet statistically significant decrease over time. In contrast, the increase in cancer mortality among women and both sexes combined has been minor and not statistically significant. Among all common malignancies, women had higher rate of gallbladder and thyroid cancer mortality than men; meanwhile, a yearly significant increase of pancreatic cancer mortality was seen among both sexes, with higher increase in women.
**Relevance**
This estimation-based study might be a substitute for constructing precise and efficient health care infrastructure to acquire better cancer control programs in India in the absence of a national cancer registry or countrywide cancer mortality data.

The cancer mortality rates are remarkably heterogeneous in the world. India has a 63.1 per 100,000 age-standardized mortality rate (ASMR) for cancer, with men and women accounting for 65.4 and 61.0, respectively. This figure is approximately half of the global ASMR of 100.7 per 100,000 for cancer (84.2 in women and 120.8 in men). The mortality rate was declining considerably for several cancers in high-income countries and increasing in low-middle–income countries, including India, because of Westernization and changes in people's lifestyle behaviors and dietary practices.^2,6,7^ Globocan estimated that 850,000 cancer deaths occurred in India in 2020. However, 28 PBCRs in India alone recorded 139,646 cancer deaths between 2012 and 2016, whereas the representing population made up only 10% of the population.^5^ This study aimed to analyze the cancer mortality trend for 23 major cancers and briefly for other leading cancer sites among men and women.

## MATERIALS AND METHODS

An estimate of nationwide cancer mortality data in India from 2000 to 2019 was obtained for 23 major cancers from the Global Health Observatory (GHO), a wing of the WHO, which provides the latest data on deaths and disability due to all causes worldwide.^7^ The data contain the number of cancer deaths in each WHO-classified age group with the corresponding population for that year. We computed the age-specific mortality rates and standardized them to the world standard populations to obtain ASMR; we used a similar method to calculate incidence rates. Using the joinpoint regression software version 4.9.1.0, we analyzed mortality trends for 23 major types of cancers between 2000 and 2019. The joinpoint program used the best fit piecewise continuous log-linear model to analyze the mortality trend by performing a sufficient number of permutations to obtain a minimum number of joinpoints. The average annual percentage change (AAPC) values are selected without inflection point for the better male and female mortality trend comparison. There were 4,499 permutations with a level of significance of 0.05 and a 95% CI for the data set. The estimated mortality trend with annual percentage change (APC) for each joinpoint, the lower and upper confidence intervals, *P* values, and AAPC for all cancers were displayed, as were graphs for men, women, and both sexes.

## RESULTS

The GHO estimated that 12.85 million deaths occurred in India between 2000 and 2019 due to 23 major cancers, and 76.3% of deaths were caused by the leading 10 cancers. Table 1 shows the estimated number of cancer mortalities in their ranking order and male-to-female ratios. Of all cancer deaths, those caused by mouth and oropharynx cancer ranked first (with 15.6%), followed by cancers of the stomach (10.6%), lung (9.6%), breast (9%), and colorectum (8%). An overall male-to-female cancer mortality ratio of 1.1 was observed in India for the past two decades. The cancer mortality was high among men than women for all common cancers except thyroid (0.6) and gallbladder (0.6) cancers. The larynx cancer had almost a 6-fold high mortality among men than women, followed by lung (2.9), melanoma (2.5), urinary bladder (2.3), mouth and oropharynx (2.2), and liver (1.9), while stomach and colorectal cancer mortality was relatively similar among both sexes

**TABLE 1:**
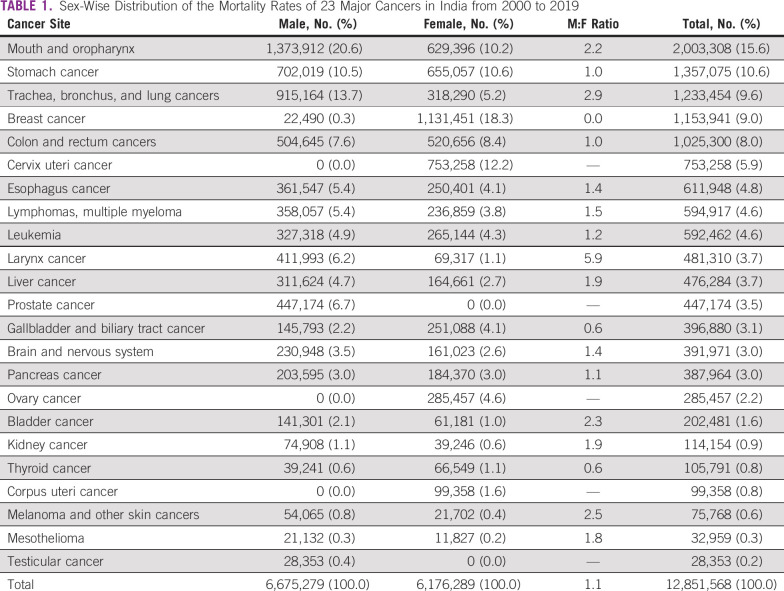
Sex-Wise Distribution of the Mortality Rates of 23 Major Cancers in India from 2000 to 2019

**FIG 1:**
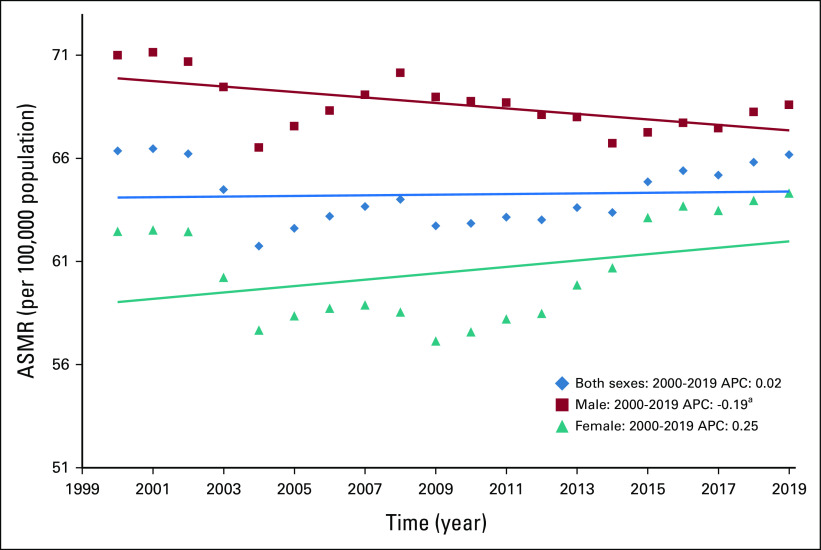
ASMR trends for 23 major cancers in India between 2000 and 2019. ^a^Signifies that the APC is significant. APC, annual percentage change; ASMR, age-standardized mortality rate.

Table 2 shows the results of the joinpoint trend analysis for 23 cancers, both site-wise and overall. The APC on the basis of ASMR during the studied years (2000-2019) decreased annually by 0.19% among men and increased nonsignificantly by 0.25% per year among women; an annual increase of 0.02% was also observed for both sexes combined (Table 2, Fig 1). However, the mortality trend varied by cancer site, and it is difficult to decipher cancer of the mouth and oropharynx because of their similar APC values overall (2000-2019) and in the first (2000-2009) and second (2010-2019) periods among men (Data Supplement [Supplemental Fig 1]). Among women, lung cancer mortality increased in the overall (2.1% per year), first (0.7% per year), and second (3.6% per year) periods. Meanwhile, mouth and oropharynx cancer cases decreased annually by 1.3% in the first period and rose by 1.2% in the second period (Table 3).

**TABLE 2:**
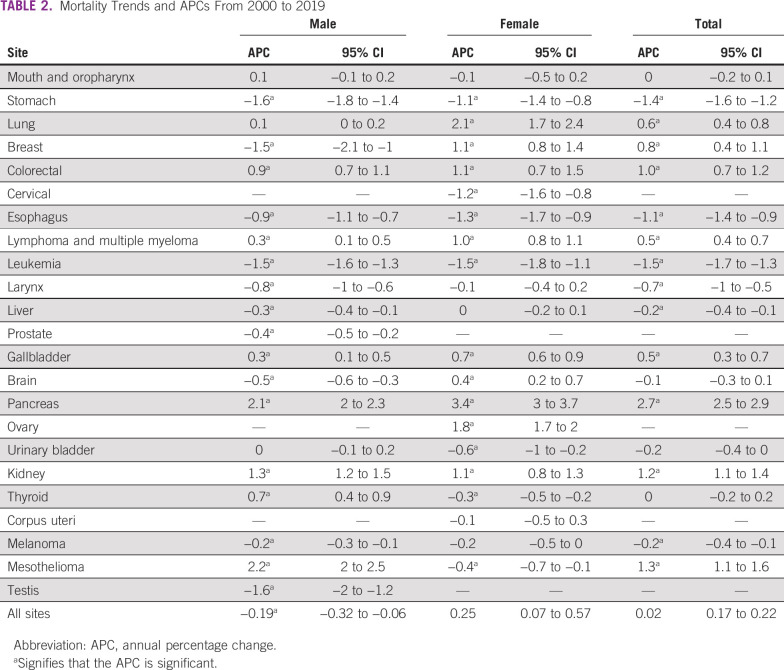
Mortality Trends and APCs From 2000 to 2019

**TABLE 3:**
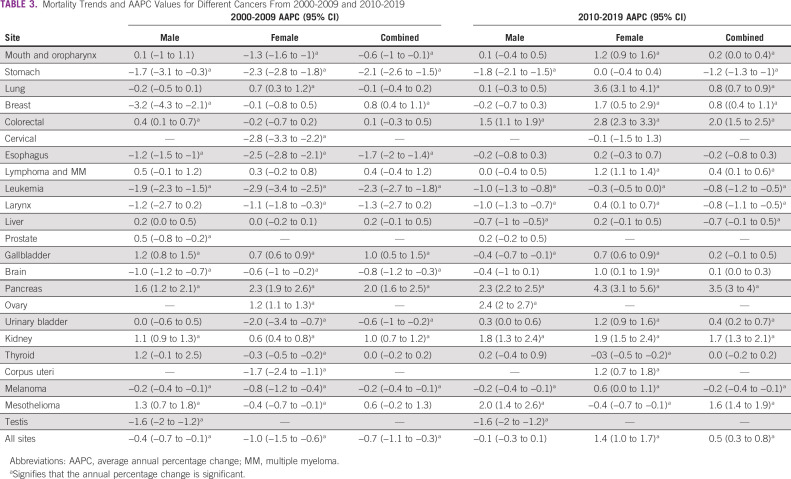
Mortality Trends and AAPC Values for Different Cancers From 2000-2009 and 2010-2019

The cancer mortality trend increased for cancers of the lung, breast, colorectum, lymphoma and multiple myeloma, gallbladder, pancreas, kidney, and mesothelioma between 2000 and 2019 (Data Supplement [Supplemental Figs 3-5, 8, 13, 15, 18, and 22]).The APC values for the aforementioned cancers were 0.6%, 0.8%, 1.0%, 0.5%, 0.5%, 2.7%, 1.2%, and 1.3%, respectively (Table 2). The highest APC in mortality was observed in pancreas cancer among combined sexes (2.7% per year) and separately for men (2.1% per year) and women (3.7% per year). Similarly, in the first and second periods, pancreatic cancer had the highest APC, whereas in the past decade, the APC rose to an average of 3.5% per year for combined sexes, 2.35% for men, and 4.3% for women (Table 3, Data Supplement [Supplemental Fig 15]).

Similar significant increasing trends in APC and AAPC were observed in colorectal, lymphoma and multiple myeloma, and kidney cancers in the total study period and the two separate periods (Tables 2 and 3, Data Supplement [Supplemental Figs 5, 8, and 18]). Meanwhile, cancers of the stomach, esophagus, leukemia, larynx, and melanoma showed a declining cancer mortality trend irrespective of sex (Data Supplement [Supplemental Figs 2, 7-11, and 21]). In the second period, the mortality trend among men continued to decrease, while a significant increasing trend was shown by these cancers, except that AAPC remained stable for stomach cancer among women.

Of the 23 investigated cancer sites, cancer of the thyroid and gallbladder had higher ASMR values among women than among men and both sexes. Breast cancer mortality among men decreased by 1.5% per year, while it increased among women by 1.1% per year (Data Supplement [Supplemental Fig 4]). The trend in mortality rose by 1.7% per year in the second period. Similarly, gallbladder cancer mortality showed an increasing trend overall (2000-2019) and in the first (2000-2009) period among men (0.3% and 1.2% per year) and women (0.7% and 0.7% per year).Meanwhile, in the second period, an increasing trend among women (by 0.7% per year) and decreasing APC (by 0.4%) per year among men (Data Supplement [Supplemental Fig 13]) were observed. However, thyroid cancer showed an increasing trend among men in all periods, while a stable annual decrease of 0.3% was seen among women (Data Supplement [Supplemental Fig 19]). Brain and liver cancer mortality trends decreased among men and increased significantly in women, whereas urinary bladder and mesothelioma decreased among women and increased in men (Data Supplement [Supplemental Figs 11, 14, 17, and 22]).

Female cancers of the cervix, corpus uteri, ovary, and breast contributed to 17.6% of deaths, while male cancers of the prostate and testis contributed to only 3.7%. The mortality trend of these cancers, except ovary and breast cancer, declined over time. We observed an annual increase of 1.8% and 1.1% in ovary and breast cancer, respectively. Moreover, in the second period, corpus uteri and ovary showed annual increases of 1.2% and 2.4%, respectively, while cervix cancer maintained a nonsignificant decreasing mortality trend of 0.1% per year (Data Supplement [Supplemental Figs 6, 16, and 20]). The mortality trend of prostate and testis cancers showed an overall decreasing trend in the overall period (with 0.4% and 1.6% APC). Meanwhile, in the first and second periods, prostate cancer showed an increasing trend with an AAPC of 0.5% and a nonsignificant APC of 0.2%. Meanwhile, testis cancer mortality substantially decreased by 1.6% per year (Data Supplement [Supplemental Figs 12 and 23]).

## DISCUSSION

To our knowledge, this is the first national-level cancer mortality trend analysis on the basis of 23 major cancer site mortality data from GHO between 2000 and 2019. Overall, we found a decreasing cancer mortality trend among men and a slight nonsignificant increase among women and among both sexes over the 20-year study period. The heterogeneity in mortality trends and poor prognosis suggest that changes are needed in cancer-causing behaviors in the population, including lifestyle habits and dietary practices, as well as in the health care system and cancer awareness.^8,9^ However, some specific cancers such as cancers of lung, breast, colorectum, lymphoma, multiple myeloma, gallbladder, pancreas, kidney, ovary, and mesothelioma showed substantial increases in mortality in India. Conversely, cancers of the stomach, esophagus, leukemia, larynx, liver, brain, testis, prostate, cervix, corpus uteri, and melanoma decreased over time. Finally, the APC values were stable for cancers of the mouth, oropharynx, and thyroid. These findings corroborate other researchers' observations, especially those reported in the Global Burden of Disease study in India.^9^

The reduction in smoke/smokeless tobacco and household air pollution could have resulted in a decrease in esophagus, lung, mouth and oropharynx, and larynx cancer incidences and may have resulted in reduced cancer mortality across the country.^9^ Low-dose computed tomography of the chest is an optimal strategy for screening and reducing mortality of lung cancer. However, it is not an optimal solution in India because of the country's relatively limited health infrastructure and the technique's high cost, high false-positivity rate, and logistic constraints.^10^

For oral cancer, screening by visual inspection can effectively reduce mortality, which was proven by a study from Kerala on oral cancer. In addition, repeated screening can reduce the oral cancer burden even more effectively.^9,11,12^ Similarly, a substantial decrease in stomach cancer mortality was observed across the country during our 20-year study period. This may be due to lifestyle changes, increase in the consumption of fruits, reduced use of salt-preserved foods, increased use of refrigeration, and reduced tobacco consumption.^9,13,14^

Furthermore, we observed that cancers of the breast, thyroid, and gallbladder had a higher mortality rate in women than men. Sex disparities in cancer signify risk factors linked to lifestyle, environment, genetics, and epigenetics.^15,16^ The consistent increase in India in breast cancer mortality among women might be due to changes in risk factors, including older age at first birth, lower parity, and being overweight or obese.^9^

Gallbladder cancer is highly lethal, and the mortality rate is higher among women than men in India and the world. The common risk factors among both sexes are geographical area, obesity, family history of gallstones, genetic risk, chronic Salmonella typhi bacterial infections, and lifestyle factors. Studies found that irregular and longer menstrual cycles, older age at first pregnancy, and higher number of pregnancies were associated with an increased risk of gallbladder cancer. These findings suggest that female hormones may contribute to the etiology of gallbladder cancer, which may explain why the incidence and mortality rate are higher in women than in men. In addition, the risk can increase by up to 12 times because of chronic Salmonella typhi bacterial infections and is twice more common in women than in men. More studies are needed to characterize the influential risk factors of gallbladder cancer.^17-23^

The growing body of research on the burden of cancer in India reported that the mortality trend has been declining for infection-related cancers, such as cervical and stomach cancers, while increasing for cancers of the breast, colorectum, and prostate, which were associated with risk factors related to lifestyle habits and aging.^9,24,25^ Meanwhile, we observed infection-related cancer of liver mortality was declining over time, while increasing for lymphomas in Indian population.

In our study, the leukemia mortality trend decreased over time, most likely due to the introduction of better treatment protocols and improvements in supportive care (availability of blood components, growth factor support, and better antibiotics). Such advancements have made it easier to identify the risk factors associated with leukemia, thus helping to reduce the leukemia burden.^26-29^

In India, more than 70% of cases of cancer are diagnosed at advanced stages because of decreased cancer awareness and lack of organized breast, oral, or cervical cancer screening programs. This fact explains the high cancer mortality rate. A nationally representative survey found that cancer deaths in India were two times higher among illiterate people than among people who had at least secondary education. Furthermore, lifestyle-related cancer mortality is higher in urban areas than in rural areas in India, whereas infection-related cancer mortality is higher in rural areas. Cancer morbidity and mortality can be reduced by appropriate changes in lifestyle habits, cancer literacy, screening, early diagnosis, and health care access.^7,30,31^

Prevention through screening and awareness could reduce the burden of preventable cancers worldwide and in India since more than 30% of cancer deaths are due to modifiable risk factors, including tobacco and alcohol use.^30^ India launched the first national screening program for breast, cervical, and oral cavity cancers, which account for 34% of all cancers in people older than 30 years in 100 districts before expanding to other areas. Screening programs are conducted once every five years through clinical breast examinations, visual inspections with acetic acid, and oral visual examinations.^30,32^

There are no standardized screening programs for other deadliest cancers in India. Studies have proved that absence of screening program responsible for a significant percentage of colorectal cancer deaths. A study from United States reported that an increase in colorectal cancer screening program to 80% would reduce incidence by 17% and mortality by 19%.^33^ However, studies have shown that a lack of knowledge about cancer symptoms causes patients to delay seeking treatment for preventable cancers. Optimal infrastructure and human resources for awareness and prevention of cancer are required alongside the strengthening of existing screening programs for cancers of the breast, cervix, and oral cavities to focus on other deadly cancers in India.^9,30,34,35^

The cancer mortality recordings in India have many limitations, mainly because of the unavailability of a nationwide cancer registration system, and incomplete and imprecise medical cause of death certificates. According to the certification of cause of death by the office of the general registrar, only 19% of deaths are medically certified in India.^5,24,36^ The mortality data of the 23 major cancers investigated in this study were based on GHO data collected between 2000 and 2019; these are estimates. Heterogeneity is often seen between statistical models and cancer registry data–based estimations, which is not surprising because of the real-time unavailability of data. For example, the cancer mortality projection for India reports that cancer mortality will increase to 0.38 million among men and 0.32 among women in 2026, but the GHO data from 2019 already exceed these values, with 0.42 million cancer deaths estimated among men and women, separately.^30,37^ By contrast, these findings provide a comprehensive understanding of the nation's most lethal disease for improving health initiatives. This exemplifies the importance of developing a pertinent strategy to gather accurate cancer data in the future for successful planning and acquiring better cancer control programs.

In conclusion, the findings show the mortality trends for 23 major cancer types by sex on the basis of 12.85 million cancer deaths in India estimated from GHO between 2000 and 2019. High-quality cancer surveillance and mortality data recording systems are needed to gain real-time data showing how cancer mortality has changed over time across India. A multifaceted strategy is required to tackle the rising cancer mortality rates in India; the best long-term strategy could be implementing awareness on cancer symptoms among the population as well as cancer prevention policies with improved health infrastructure and specifically dedicated human resources.
